# Supplementary material for: Sex estimation techniques based on skulls in forensic anthropology: A scoping review
Source: PLoS One. 2024 Dec 9;19(12):e0311762. doi: 10.1371/journal.pone.0311762 (PMC11627412; doi:10.1371/journal.pone.0311762)
Supplement: S2 Table — (DOCX) [file pone.0311762.s002.docx]

**Table S2: Characteristics of the included articles**

**List of Abbreviations**

| **Abbreviations** | **Definition** |
| --- | --- |
| ANN | Artificial Neural Networks |
| AP | Anteroposterior |
| BLR | Binary Logistic Regression |
| CBCT | Cone Beam Computed Tomography |
| DFA | Discriminant Function Analysis |
| DL | Deep Learning |
| DT | Decision Tree |
| ETC | Extra Tree Classifier |
| GBDT | Gradient Boosting Decision Tree |
| ICC | Intraclass Correlation Coefficient |
| KNN | K-Nearest Neighbor |
| LCs | Lateral Cephalograms |
| LGB | Light Gradient Boosting |
| LR | Logistic Regression |
| MARS | Multivariate Adaptive Regression Splines |
| MLR | Multinomial Logistic Regression |
| MRI | Magnetic Resonance Imaging |
| MSCT | Multi-Slice Spiral Computed Tomography |
| NBC | Naive Bayes Classification |
| OPG | Orthopantomograms |
| PA | Postero‑Anterior |
| PMCT | Post-Mortem Computed Tomography |
| PMLR | Penalized Multinomial Logistic Regression |
| QDA | Quadratic Discriminant Analysis |
| RF | Random Forest |
| SMV | Submentovertex |
| SVM | Support Vector Machine |
| XGB | Extreme Gradient Boosting |
|  |  |

**Supplementary Table 1. Non-metric studies**

| Reference | Population | Anatomical Site | Sample Size | Modality | Analysis Model | Inter/Intra Bias | Training/Testing Accuracy (%) |
| --- | --- | --- | --- | --- | --- | --- | --- |
| Yaşar et al. (2023) [76] | Turkish | Walker | 142 (training); 50 (testing) | dry skulls | LR | Kappa: 0.52~0.74/0.60~0.83 | 91.5/90.0 |
| Yang et al. (2023) [87] | Chinese | Walker | 486 (training); 90 (testing) | 3D-MSCT | LR | Kappa: 0.689/0.874 | 82.9/73.0 |
| Alves et al. (2020) [104] | Brazilian | Walker | 179 | dry skulls | LR | —/— | 87.4/— |
| Jeong et al. (2022) [107] | Korean | mastoid | 100 | 3D-MSCT | LR | —/— | 50.0/— |
| taek et al. (2023) [111] | Korean | Walker | 135 | 3D-PMCT | DFA, DT | Kappa: —/0.60~0.79 | DFA: 88.1/—; DT: 90.4/— |
| Rogers et al. (2024) [112] | South Asian | Walker | 34 | dry skulls | DFA | Kappa: —/0.298~0.468 | 63.3/— |
| Cappella et al. (2022) [113] | Italian | Walker | 124 (training); 53 (testing) | dry skulls | LR | Kappa: 0.73~0.86/0.70~0.96 | 91.1/92.4 |
| Bareša et al. (2024) [114] | Croatian | Walker | 280 | 3D-MSCT | LR | Kappa: 0.643~0.815/0.859~0.942 | 86.3/— |
| Nikita et al. (2020) [115] | Greek | Walker | 132 (training); 59 (testing) | dry skulls | BLR, MLR, PMLR, DFA, NBC, DT, RF, ANN, SVM, MARS, XGB | —/— | BLR: 94.1/84.4; MLR: 94.1/84.4; pMLR: 92.4/82.8; DFA: 92.4/86.2; NBC: 92.4/87.9; DT: 93.3/82.8; RF: 100/79.3; ANN: 92.4/84.5; SVM: 93.3/86.2; MARS: 93.3/82.8; XGB: 93.3/82.8 |

**Supplementary Table 2. Traditional metric studies**

| Reference | Population | Anatomical Site | Sample Size | Modality | Analysis Model | Inter/Intra Bias | Training/Testing Accuracy (%) |
| --- | --- | --- | --- | --- | --- | --- | --- |
| Ningtyas et al. (2023) [33] | Indonesian | frontal bone | 108 (training); 30 (testing) | LCs | DFA | ICC: 0.769~0.972/0.931~0.977 | 75.9/70.0 |
| Meral et al. (2020) [35] | Turkish | foramen magnum | 600 | 2D-MSCT | DFA | —/— | 75.0/— |
| Toneva et al. (2021) [36] | Bulgarian | cranium | 393 | 3D-MSCT | SVM, LR, ANN | ICC: 0.725~1.000/0.752~0.997 | SVM: 96.1/—; LR: 95.5/—; ANN: 95.2/— |
| Cekdemir et al. (2021) [37] | Caucasian | cranium | 616 | 3D-MSCT | LR | Kappa: 0.82/— | 94.7/— |
| Techataweewan et al. (2021) [43] | Thailand | cranium | 322 | dry skulls | DFA | —/— | 92.1/— |
| Deshpande et al. (2022) [58] | Indian | maxillary sinus | 60 | 2D-CBCT | DFA | —/— | 71.3/— |
| Denny et al. (2023) [59] | Indian | frontal sinus, mandible | 142 | 2D-CBCT | DFA | —/— | frontal sinus: 66.9/—; mandible: 68.4/— |
| Girdhar et al. (2023) [60] | Indian | mandible | 191 | LCs | LR | —/— | 60.7/— |
| Jacob et al. (2022) [61] | Indian | occipital condyles | 86 | dry skulls | DFA | —/— | 66.3/— |
| Kannampurath et al. (2023) [62] | Indian | maxillary sinus | 200 | 2D-CBCT | DFA | —/— | 69.5/— |
| Kumar et al. (2023) [63] | Indian | cranium | 217 | 2D+3D-MSCT | DFA | ICC: 0.965~0.999/0.936~0.999 | 93.6/— |
| Kurniawan et al. (2023) [64] | Indian | maxillary sinus | 130 | LCs | DFA | Cronbach's alpha: —/0.994 | 76.2/— |

**Supplementary Table 2 (continued)**

| Reference | Population | Anatomical Site | Sample Size | Modality | | Analysis Model | Inter/Intra Bias | Training/Testing Accuracy (%) |
| --- | --- | --- | --- | --- | --- | --- | --- | --- |
| Mathew et al. (2020) [65] | Indian | Maxillary Sinus | 100 | 2D-CBCT | | DFA | ICC: —/0.83~0.96 | 79.0/— |
| Mondal et al. (2022) [66] | Indian | mastoid | 60 | 2D-CBCT | | DFA | —/— | 76.7/— |
| Passey et al. (2021) [67] | Indian | mastoid | 100 | lateral radiographs | | DFA | —/— | 78.0/— |
| Ramamoorthy et al. (2020) [68] | Indian | cranium | 70 | 2D-MSCT | | DFA | —/— | 91.4/— |
| Saini et al. (2022) [69] | Indian | mandible | 385 | dry skulls | | DFA | —/— | 84.7/— |
| Sathawane et al. (2022) [70] | Indian | skull | 200 | PA cephalograms | | DFA | —/— | 88.5/— |
| Verma et al. (2021) [71] | Indian | foramen magnum | 100 | SMV radiographs | | DFA | agreement: 85%/— | 69.7/— |
| Komut et al. (2021) [72] | Turkish | crista galli | 533 | 2D-MSCT | | LR | —/— | 88.6/— |
| Meral et al. (2022) [73] | Turkish | cranium | 600 | 3D-MSCT | | DFA | ICC: 0.85~0.98/0.87~0.99 | 88.0/— |
| Meral et al. (2022) [74] | Turkish | orbital region | 600 | 3D-MSCT | | DFA | ICC: 0.75~0.87/0.75~0.96 | 73.0/— |
| Toy et al. (2022) [75] | Turkish | cranium | 300 | 2D-MSCT | LR, DFA, QDA, RF, ETC, DT | | —/— | LR: 87.8/—; DFA: 87.7/—; QDA: 86.5/—; RF: 85.8/—; ETC: 77.2/—; DT: 80.3/— |
| Emekli (2023) [77] | Turkish | frontal sinus | 350 | AP radiographs | DFA | | —/— | 69.7/— |
| Golpinar et al. (2022) [78] | Turkish | crista galli | 207 | dry skulls | | LR | —/— | 94.2/— |

**Supplementary Table 2 (continued)**

| Reference | Population | Anatomical Site | Sample Size | Modality | Analysis Model | Inter/Intra Bias | Training/Testing Accuracy (%) |
| --- | --- | --- | --- | --- | --- | --- | --- |
| Farhadian et al. (2020) [79] | Iranian | mastoid | 171 (training); 19 (testing) | 3D-CBCT | KNN, ANN, NB, RF, LR, DFA, SVM | —/— | KNN: 91.9/90.9; ANN:84.1/84.7; NB: 93.1/92.5; RF: 97.5/96.9; LR: 92.5/91.7; DFA: 92.3/91.9; SVM: 93.7/92.7 |
| Banihashem et al. (2023) [80] | Iranian | sphenoid sinus | 469 | 2D-CBCT | DFA | —/— | 90.2/— |
| Rad et al. (2020) [81] | Iranian | mandible | 532 | OPG | DFA | —/— | 82.5/— |
| Salemi et al. (2021) [82] | Iranian | mastoid | 190 | 3D-CBCT | DFA | ICC: 0.935~0.955/0.945 | 92.6/— |
| Salmanipour et al. (2023) [83] | Iranian | cranium | 199 | 2D-MSCT | LR, GBDT | —/— | LR: 0.83/—; GBDT: 0.89/— |
| Liu et al. (2023) [85] | Chinese | sphenoid sinus | 79 | MRI | DFA | —/— | 63.3/— |
| Jaafar et al. (2022) [98] | Nigeria | foramen magnum, occipital condyles | 399 | 2D-MSCT | DFA | —/— | 54.4/— |
| Ominde et al. (2022) [99] | Nigeria | foramen magnum | 336 | 2D-MSCT | DFA | ICC: 0.511~0.583/0.530~0.551 | 71.4/— |
| Ominde et al. (2023) [100] | Nigeria | maxillary sinus | 292 | 2D-MSCT | DFA | —/— | 87.3/— |
| Abo et al. (2020) [102] | Egyptians | foramen magnum; occipital condyles | 367 | 2D-MSCT | LR | —/— | 70.9/69 |
| Sassi et al. (2020) [103] | Brazilian | cranium | 320 | dry skulls | LR, Ctree | —/— | LR: 78.0/—; Ctree: 70.3/— |
| Berezowski et al. (2021) [105] | American | cranium | 91 | photogrammetry | DFA | —/— | 90.0/75.0 |

**Supplementary Table 2 (continued)**

| Reference | Population | Anatomical Site | Sample Size | Modality | Analysis Model | Inter/Intra Bias | Training/Testing Accuracy (%) |
| --- | --- | --- | --- | --- | --- | --- | --- |
| Kresic et al. (2023) [108] | Croatian | orbital region | 414 | 3D-MSCT | DFA | —/— | 73.5/— |
| Petaros et al. (2021) [109] | Croatian | mastoid | 170 | 3D-MSCT | DFA | coefficient of variability: —/0.00%~51.10% | 74.1/— |
| Uabundit et al. (2021) [118] | Thailand | pterion | 124 | dry skulls | RF | —/— | 80.7/— |
| Nikita et al. (2024) [119] | Greek | cranium | 227 | dry skulls | XGB, LGB; DFA | ICC: —/0.393~0.987 | XGB: 71.3/—; LGB: 73.5/—; DFA: 55.7/— |
| Packirisamy et al. (2024) [123] | Saudi Arabian | orbital region | 456 | 3D-MSCT | DFA | —/— | 92.1/— |
| Cappella et al. (2020) [124] | Italian | cranium, mandible | 80 | dry skulls | DFA | agreement: 94%/97% | cranium: 88.6/— ; mandible: 74.7/— |
| Abualhija et al. (2020) [125] | Jordanian | mandible | 50 | OPG | LR | ICC: 0.707~0.991/0.885~0.999 | 77.6/80 |
| Aljarrah et al. (2022) [126] | Saudi Arabian | foramen magnum, occipital condyles | 472 | 2D-CT | DFA | ICC: 0.89~0.99/— | 71.6/— |
| Atreya et al. (2023) [127] | Nepalese | foramen magnum, occipital condyles | 261 | 2D-MSCT | DFA | ICC: 0.853/0.876 | 71.0/— |
| Soon et al. (2023) [128] | Malaysian | foramen magnum | 300 | 2D-PMCT | DFA | —/— | 60.0/— |

**Supplementary Table3. GM and DL studies**

| Reference | Population | Anatomical Site | Sample Size | Modality | Analysis Model | Inter/Intra Bias | Training/Testing Accuracy (%) |
| --- | --- | --- | --- | --- | --- | --- | --- |
| Sobhani et al. (2021) [84] | Iranian | mastoid | 80 | 3D-CBCT | DFA | —/— | 88.8/— |
| Yang et al. (2020) [86] | Chinese | supraorbital margin; skull contour | 93 (training); 40 (testing) | 3D-MSCT | SVM | —/— | —/92.6 |
| Aida et al. (2020) [88] | Bosnian | occipital condyles | 214 | laser scanner | DFA | —/— | 64.6/— |
| Ajanović et al. (2023) [89] | Bosnian | orbital region | 211 | laser scanner | DFA | —/— | 87.6/— |
| Ajanovic et al. (2022) [90] | Bosnian | hard palate | 209 | laser scanner | DFA | —/— | 66.3/— |
| Ajanovic et al. (2023) [91] | Bosnian | foramen magnum | 214 | laser scanner | DFA | —/— | 64.1/— |
| Sarač-Hadžihalilović et al. (2022) [92] | Bosnian | pyriform aperture | 211 | laser scanner | DFA | —/— | 67.4/— |
| Toneva et al. (2021) [93] | Bulgarian | cranium | 393 | 3D-MSCT | DT | ICC: 0.609~0.997/0.725~1.000 | 91.4/— |
| Toneva et al. (2022) [94] | Bulgarian | cranium | 373 | 3D-MSCT | DFA, k-means clustering | ICC: 0.884~0.928/0.986~0.993 | DFA: 92.9/—; k-means clustering: 86.3/— |
| Toneva et al. (2023) [95] | Bulgarian | mandible | 190 | 3D-MSCT | DFA | landmark digitization error: —/<1mm | 87.4/— |
| Toneva et al. (2022) [96] | Bulgarian | cranium | 340 | 3D-MSCT | DFA | ICC: —/0.997 | 92.9/— |

**Supplementary Table3. (continued)**

| Reference | Population | Anatomical Site | Sample Size | Modality | Analysis Model | Inter/Intra Bias | Training/Testing Accuracy (%) |
| --- | --- | --- | --- | --- | --- | --- | --- |
| Boucherie et al. (2022) [97] | Western Europe | occipital condyles; mastoid | 50 | laser scanner | DFA | —/— | 82.0/— |
| Meinerová et al. (2023) [101] | Egyptians; ancient Egyptians | cranium | 96/54 | Egyptians: 3D-MSCT; ancient Egyptians: laser scanner | SVM | —/— | 89.6/—; 61.1/— |
| Kondou et al. (2023) [106] | Japanese | skull | 988 (training); 246 (testing) | 3D-PMCT | DL | —/— | 95.0/93.0 |
| Čechová et al. (2021) [110] | Czech | frontal bone | 103 | 3D-MSCT | SVM | —/— | 72.8/— |
| Imaizumi et al. (2020) [116] | Japanese | skull | 100 | 3D-PMCT | SVM | —/— | skull: 90.6/—; cranium: 90.7%/——; mandible: 84.1/— |
| Bertsatos et al. (2020) [117] | Czech; Greek | cranium | Czech:170; Greek:156. 223 (training); 103 (testing) | Czech:3D-MSCT; Greek: photogrammetry | DFA, SVM | —/— | DFA: 96.7/85.4; SVM: 92.2/82.4 |

—: Not Report

The citation numbers in Table S2 match those listed in the reference section of the main text.

**References**

1. Ningtyas AH, Widyaningrum R, Shantiningsih RR, Yanuaryska RD. Sex estimation using angular measurements of nasion, sella, and glabella on lateral cephalogram among Indonesian adults in Yogyakarta. Egypt J Forensic Sci. 2023;13(1):48. doi: 10.1186/s41935-023-00368-9.
2. Meral O, Belkıs Toklu B, Meydan R, Kaya A, Karadayı B, Acar T. Sex estimation from foramen magnum parameters in adult Turkish population: A computed tomography study. Legal Med-Tokyo. 2020;47:101775. doi: 10.1016/j.legalmed.2020.101775.
3. Toneva DH, Nikolova SY, Agre GP, Zlatareva DK, Hadjidekov VG, Lazarov NE. Data mining for sex estimation based on cranial measurements. Forensic Sci Int. 2020;315:11044. doi: 10.1016/j.forsciint.2020.110441.
4. Cekdemir YE, Mutlu U, Karaman G, Balci A. Estimation of sex using morphometric measurements performed on cranial computerized tomography scans. Radiol Med. 2021;126(2):306-15. doi: 10.1007/s11547-020-01233-8.
5. Techataweewan N, Hefner JT, Freas L, Surachotmongkhon N, Benchawattananon R, Tayles N. Metric sexual dimorphism of the skull in Thailand. Forensic Sci Int: Rep. 2021;4:100236. doi: 10.1016/j.fsir.2021.100236.
6. Deshpande AA, Munde AD, Mishra SS, Kawsankar KD, Sawade RV, Mandar B. Determination of sexual dimorphism of maxillary sinus using cone-beam computed tomography in a rural population of western Maharashtra- A retrospective, cross-sectional study. J Family Med Prim Care. 2022;11(4):1257-61. doi: 10.4103/jfmpc.jfmpc_389_21.
7. Denny C, Bhoraskar M, Abdul Aziz Shaikh S, T S B, Sujir N, Natarajan S. Investigating the link between frontal sinus morphology and craniofacial characteristics with sex: A 3D CBCT study on the South Indian population. F1000Research. 2023;12:811. doi: 10.12688/f1000research.137008.2.
8. Girdhar A, Keerthika R, Narwal A, Kamboj M, Devi A, Sharma R. Comparative manual and digital analysis of gonial angle in lateral cephalograms for gender determination. Forensic Sci Med Pat. 2024;20(1):73-8. doi: 10.1007/s12024-023-00625-2.
9. Jacob M, Bindhu S. Morphometric assessment methods for estimation of sexual dimorphism from cranial occipital condyles. Int J Morphol. 2022;40(4):1128-33. Doi: 10.4067/S0717-95022022000401128.
10. Kannampurath A, Srikantannair SL, Mathew P, Sivaprasad T. Maxillary sinus in gender determination: a morphometric analysis using cone beam computed tomography. Forensic Sci Med Pathol. 2023 Nov12. doi: 10.1007/s12024-023-00749-5.
11. Kumar Battan S, Sharma M, Gakhar G, Garg M, Singh P, Jasuja OP. Cranio-facial bones evaluation based on clinical CT data for sex determination in Northwest Indian population. Legal Med-Tokyo. 2023;64:102292. doi: 10.1016/j.legalmed.2023.102292.
12. Kurniawan A, Athalia SA, Prakoeswa BFWR, Rizky BN, Chusida A, Yudha M, et al. Cephalometric radiograph-based approach for sex determination using maxillary sinus index in Surabaya, Indonesia. Eur J Anat. 2023;27(6):663-8. doi: 10.52083/DHPM3206.
13. Mathew A, Jacob L. 3D evaluation of maxillary sinus in gender determination: A cone beam computed tomography study. J Indian Acad Oral M. 2020;32(4):384-9. doi: 10.4103/jiaomr.jiaomr_104_20.
14. Mondal B, Vaishali MR, David MP, Roopashri G, Kumar V, Ponnuswamy IA. Assessment of the usefulness of morphometric and volumetric analysis of mastoid process for gender determination in forensic odontology: A retrospective cone beam computed tomographic study. J Indian Acad Oral M. 2022;34(1):82-6. doi: 10.4103/jiaomr.jiaomr_229_21.
15. Passey J, Pandey S, Passey N, Singh R, Singh R, Kumar A. Radiographic Evaluation of Mastoid Parameters for Sexual Differentiation in North Indian Population. Cureus. 2021;13(6):e16011. doi: 10.7759/cureus.16011. PubMed PMID: 34336501.
16. Ramamoorthy B, Pai MM, Ullal S, Prabhu LV. Discriminant function analysis of craniometric traits for sexual dimorphism and its implication in forensic anthropology. J Anat Soc India. 2019;68(4):260-8. doi: 10.4103/JASI.JASI_82_19.
17. Saini V, Chowdhry A, Mehta M. Sexual dimorphism and population variation in mandibular variables: a study on a contemporary Indian population. Anthropol Sci. 2022;130(1):59-70. doi: 10.1537/ase.2108282.
18. Sathawane R, Tripathi S, Sukhadeve V. Introduction of a new parameter 'Mastoid to Gonion Height' and its comparative evaluation with established pa cephalometric parameters in sex determination: An analytical study. J Indian Acad Oral M. 2022;34(2):218-22. doi: 10.4103/jiaomr.jiaomr_24_22.
19. Verma P, Gupta N, Sameera Y, Faraz S, Sharma P, Sharma B. Foramen magnum as determinant of sexual dimorphism in Sri Ganganagar population: A radiographic study. J Indian Acad Oral M. 2021;33(1):71-6. doi: 10.4103/jiaomr.jiaomr_205_20.
20. Komut E, Golpinar M. A comprehensive morphometric analysis of crista galli for sex determination with a novel morphological classification on computed tomography images. Surg Radiol Anat. 2021;43(12):1989-98. doi: 10.1007/s00276-021-02799-2.
21. Meral O, Meydan R, Toklu BB, Kaya A, Karadayi B, Acar T. Estimation of sex from computed tomography images of skull measurements in an adult Turkish population. Acta Radiol. 2022;63(11):1513-21. doi: 10.1177/02841851211044978.
22. Meral O, Toklu BB, Meydan R, Kaya A, Karadayı B, Acar T. Sexing from the orbital measurements using computed tomography images and discriminant function analysis in Turkish population. Imaging Sci J. 2022;70(4):207-13. doi: 10.1080/13682199.2023.2165237.
23. Toy S, Secgin Y, Oner Z, Turan MK, Oner S, Senol D. A study on sex estimation by using machine learning algorithms with parameters obtained from computerized tomography images of the cranium. Sci Rep-UK. 2022;12(1):4278. doi: 10.1038/s41598-022-07415-w.
24. Yaşar B, Sağır M. Testing of morphological sex estimation traits with a sex-known collection: Ottoman period skulls. Int J Osteoarchaeol. 2023;33(6):1042-51. doi: 10.1002/oa.3265.
25. Emekli E. Sex determination using frontal sinus diameters on direct radiography. Cureus. 2023;15(10):e47476. doi: 10.7759/cureus.47476. PubMed PMID: 38022337.
26. Golpinar M, Salim H, Ozturk S, Komut E, Sindel M. Sex estimation with morphometric and morphological characteristics of the crista galli. Surg Radiol Anat. 2022;44(7):1007-15. doi: 10.1007/s00276-022-02971-2.
27. Farhadian M, Salemi F, Shokri A, Safi Y, Rahimpanah S. Comparison of data mining algorithms for sex determination based on mastoid process measurements using cone-beam computed tomography. Imagng Sci Dent. 2020;50(4):323-30. doi: 10.5624/ISD.2020.50.4.323.
28. Banihashem Rad SA, Anbiaee N, Moeini S, Bagherpour A. Sex determination using human sphenoid sinus in a northeast Iranian population: A discriminant function analysis. J Dent. 2023;24:95-102. doi: 10.30476/dentjods.2022.92915.1685.
29. Rad FO, Javanshir B, Nemati S, Khaksari F, Mansoori R, Ranjzad H, et al. Evaluation of sexual dimorphism with mandibular parameters by digital panoramic radiography. Open Dent J. 2020;14(1):172-7. doi: 10.2174/1874210602014010172.
30. Salemi F, Farhadian M, Shokri A, Safi Y, Rahimpanah S. Sex determination by osteometric assessment of the mastoid process using Cone Beam Computed Tomography. Brazilian Dental Science. 2021;24(1):1-9. doi: 10.14295/bds.2021.v24i1.2075.
31. Salmanipour A, Memarian A, Tofighi S, Vahedifard F, Khalaj K, Shiri A, et al. Prediction of sex, based on skull CT scan measurements in Iranian ethnicity by machine learning-based model. Foren Imag. 2023;33:200549. doi: 10.1016/j.fri.2023.200549.
32. Sobhani F, Salemi F, Miresmaeili A, Farhadian M. Morphometric analysis of the inter-mastoid triangle for sex determination: Application of statistical shape analysis. Imagng Sci Dent. 2021;51:1-8. doi: 10.5624/isd.20200297.
33. Liu HY, Bai ZY, Jing W, Yang XF, Kwon TG, Yun KM, et al. Application of sphenoid sinus morphological characteristics in sex estimation based on magnetic resonance imaging images. Int J Morphol. 2023;41(4):1166-70. doi: 10.4067/S0717-95022023000401166.
34. Yang W, Zhou MQ, Zhang PF, Geng GH, Liu XN, Zhang HB. Skull sex estimation based on wavelet transform and Fourier transform. Biomed Res Int. 2020;2020(1):8608209. doi: 10.1155/2020/8608209.
35. Yang XT, Sun CH, Ma YG, Cao YJ, Xiong J, Zhang J, et al. Sex estimation of Han adults in Western China based on three-dimensional cranial CT reconstruction. Journal of Forensic Medicine. 2023;39(1):27-33. doi: 10.12116/j.issn.1004-5619.2022.220101.
36. Aida SH, Zurifa A, Ilvana H, Senad S, Maida RT, Izeta A, et al. Bioanthropological analysis of human occipital condyles using geometric morphometric method. Saudi J Biol Sci. 2020;27(12):3415-20. doi: 10.1016/j.sjbs.2020.09.019.
37. Ajanović Z, Ajanović U, Dervišević L, Hot H, Voljevica A, Talović E, et al. A geometric morphometrics approach for sex estimation based on the orbital region of human skulls from Bosnian population. Scanning. 2023;2023(1):2223138. doi: 10.1155/2023/2223138.
38. Ajanovic Z, Dervisevic L, Dervisevic A, Sarac-Hadzihalilovic A, Dervisevic E, Tokic JB, et al. Sex prediction by geometric morphometric analysis of the hard palate. Eur Rev Med Pharmaco. 2022;26(17):6057-64. doi: 10.26355/eurrev_202209_29621.
39. Ajanovic Z, Dervisevic L, Dervisevic E, Lujinovic A, Ajanovic U, Biscevic-Tokic J, et al. Sex estimation based on foramen magnum: A three-dimensional geometric morphometrics approach. Int J Morphol. 2023;41(2):410-6. doi: 10.4067/S0717-95022023000200410.
40. Sarač-Hadžihalilović A, Ajanović Z, Hasanbegović I, Šljuka S, Rakanović-Todić M, Aganović I, et al. Analysis of gender differences on pyriform aperture of human skulls using geometric morphometric method. Folia Morphol. 2022;81(3):707-14. doi: 10.5603/FM.a2021.0080. PubMed PMID: 34608986.
41. Toneva D, Nikolova S, Agre G, Zlatareva D, Hadjidekov V, Lazarov N. Machine learning approaches for sex estimation using cranial measurements. Int J Legal Med. 2021;135(3):951-66. doi: 10.1007/s00414-020-02460-4.
42. Toneva D, Nikolova S, Tasheva-Terzieva E, Zlatareva D, Lazarov N. A geometric morphometric study on sexual dimorphism in viscerocranium. Biology-Basel. 2022;11(9):1333. doi: 10.3390/biology11091333.
43. Toneva DH, Nikolova SY, Fileva NF, Zlatareva DK. Size and shape of human mandible: Sex differences and influence of age on sex estimation accuracy. Legal Med-Tokyo. 2023;65:102322. doi: 10.1016/j.legalmed.2023.102322.
44. Toneva DH, Nikolova SY, Tasheva-Terzieva ED, Zlatareva DK, Lazarov NE. Sexual dimorphism in shape and size of the neurocranium. Int J Legal Med. 2022;136(6):1851-63. doi: 10.1007/s00414-022-02876-0.
45. Boucherie A, Chapman T, García-Martínez D, Polet C, Vercauteren M. Exploring sexual dimorphism of human occipital and temporal bones through geometric morphometrics in an identified Western-European sample. Am J Biol Anthropol. 2022;178(1):54-68. doi: 10.1002/ajpa.24485.
46. Jaafar A, Tersoo MA, Farrau U, Aliyu IS, Adamu LH, Ibrahim MZ, et al. The use of foramen magnum in the determination of sex using Computed Tomography (CT) scan images of sampled population attending National Ear Care Centre Kaduna State, Nigeria. Arab Journal of Forensic Sciences and Forensic Medicine. 2022;4(2):112-26. doi: 10.26735/LLST3350.
47. Ominde BS, Igbigbi PS. A retrospective study to evaluate the morphometry of the foramen magnum and its role in forensic science in a nigerian population of Delta State. Journal of Forensic Science and Medicine. 2022;8(2):46-51. doi: 10.4103/jfsm.jfsm_41_21.
48. Ominde BS, Ikubor JE, Iju WJ, Ebeye A, Igbigbi PS. Morphometry of the maxillary sinus and its role in sex determination in Delta State Nigeria: Retrospective CT study. Journal of Indian Academy of Forensic Medicine. 2023;45(1):52-7. doi: 10.48165/jiafm.2023.45.1.15.
49. Meinerová T, Sutoová D, Havelková PB, Velemínská J, Dupej J, Bejdová S. How reliable is the application of the sex classifier based on exocranial surface (Musilova et al., 2016) for geographically and temporally distant skull series. Forensic Sci Int. 2023;352:111850. doi: 10.1016/j.forsciint.2023.111850.
50. Abo El-Atta HMH, Abdel-Rahman RH, El-Hawary G, Abo El-Al-Atta HM. Sexual dimorphism of foramen magnum: An Egyptian study. Egypt J Forensic Sci. 2020;10(1):1. doi: 10.1186/s41935-019-0167-x.
51. Sassi C, Picapedra A, Álvarez-Vaz R, Schmidt CM, Ulbricht V, Júnior ED, et al. Sex determination in a brazilian sample from cranial morphometric parameters-a preliminary study. Journal of Forensic Odonto-Stomatology. 2020;38(1):8-17.
52. Alves N, Deana NF, González J, Hernández P, Ceballos F. Sex prediction by analysis of the morphological characteristics of macerated skulls. Int J Morphol. 2020;38(4):815-9. doi: 10.4067/S0717-95022020000400815.
53. Berezowski V, Rogers T, Liscio E. Evaluating the morphological and metric sex of human crania using 3-dimensional (3D) technology. Int J Legal Med. 2021;135(3):1079-85. doi: 10.1007/s00414-020-02305-0.
54. Kondou H, Morohashi R, Kimura S, Idota N, Matsunari R, Ichioka H, et al. Artificial intelligence-based forensic sex determination of East Asian cadavers from skull morphology. Sci Rep-UK. 2023;13(1):21026. doi: 10.1038/s41598-023-48363-3.
55. Jeong YH, Koo HN, Kim YS, Lee B, Kim S, Shim YT. Using 3D images of Korean's mastoid process to estimate sex: A metric study. Foren Imag. 2022;31:200527. doi: 10.1016/j.fri.2022.200527.
56. Kresic E, Basic Z, Jerkovic I, Kruzic I, Cavka M, Erjavec I. Sex estimation using orbital measurements in the Croatian population. Forensic Sci Med Pat. 2023;19(3):303-9. doi: 10.1007/s12024-022-00528-8.
57. Petaros A, Sholts SB, Čavka M, Šlaus M, Wärmländer SKTS. Sexual dimorphism in mastoid process volumes measured from 3D models of dry crania from mediaeval Croatia. HOMO. 2021;72(2):113-27. doi: 10.1127/homo/2021/1243.
58. Čechová M, Dupej J, Brůžek J, Bejdová Š, Velemínská J. A test of the Bulut et al. (2016) landmark-free method of quantifying sex differences in frontal bone roundness in a contemporary Czech sample. J Forensic Sci. 2021;66(2):694-9. doi: 10.1111/1556-4029.14603.
59. taek Shim Y, Kim DI, Aum N, gyu Choi S, Lee YS, Koo HN, et al. Statistical classification methods for estimating sex based on five skull traits: A nonmetric assessment using 3D CT models. HOMO. 2023;74(1):45-54. doi: 10.1127/homo/2023/1632.
60. Rogers LM, Halcrow SE, Kleffmann T, King CL. Comparing Walker's (2008) skull trait sex estimation standard to proteomic sex estimation for a group of South Asian individuals. Forensic Science International: Synergy. 2024;8:100450. doi: 10.1016/j.fsisyn.2023.100450.
61. Cappella A, Bertoglio B, Di Maso M, Mazzarelli D, Affatato L, Stacchiotti A, et al. Sexual dimorphism of cranial morphological traits in an Italian sample: A population-specific logistic regression model for predicting sex. Biology-Basel. 2022;11(8):1202. doi: 10.3390/biology11081202.
62. Bareša T, Jerković I, Jerković N, Dolić K, Dujić G, Borić MĆ, et al. Walker's traits for sex estimation in modern Croatian population using MSCT virtual cranial database: Validation and development of population-specific standards. Foren Imag. 2024;36:200578. doi: 10.1016/j.fri.2024.200578.
63. Nikita E, Nikitas P. On the use of machine learning algorithms in forensic anthropology. Legal Med-Tokyo. 2020;47:101771. doi: 10.1016/j.legalmed.2020.101771.
64. Imaizumi K, Bermejo E, Taniguchi K, Ogawa Y, Nagata T, Kaga K, et al. Development of a sex estimation method for skulls using machine learning on three-dimensional shapes of skulls and skull parts. Foren Imag. 2020;22:200393. doi: 10.1016/j.fri.2020.200393.
65. Bertsatos A, Chovalopoulou ME, Brůžek J, Bejdová Š. Advanced procedures for skull sex estimation using sexually dimorphic morphometric features. Int J Legal Med. 2020;134(5):1927-37. doi: 10.1007/s00414-020-02334-9.
66. Uabundit N, Chaiyamoon A, Iamsaard S, Yurasakpong L, Nantasenamat C, Suwannakhan A, et al. Classification and morphometric features of pterion in thai population with potential sex prediction. Medicina-Lithuania. 2021;57(11):1282. doi: 10.3390/medicina57111282.
67. Nikita PA, Garoufi N, Valakos E, Constantinou C, Nikita E, Chovalopoulou ME. Testing the accuracy of the SexEst software for sex estimation in a modern Greek sample. Int J Osteoarchaeol. 2024;34(2):e3283. doi: 10.1002/oa.3283.
68. Packirisamy V, Aljarrah K, Nayak SB. Morphometric evaluation of the orbital region for sex determination in a Saudi Arabian population using 3D CT images. Anat Sci Int. 2024;99(1):118-26. doi: 10.1007/s12565-023-00742-6.
69. Cappella A, Gibelli D, Vitale A, Zago M, Dolci C, Sforza C, et al. Preliminary study on sexual dimorphism of metric traits of cranium and mandible in a modern Italian skeletal population and review of population literature. Legal Med-Tokyo. 2020;44:101695. doi: 10.1016/j.legalmed.2020.101695.
70. Abualhija D, Revie G, Manica S. Mandibular ramus as a sex predictor in adult Jordanian subjects. Foren Imag. 2020;21:200366. doi: 10.1016/j.fri.2020.200366.
71. Aljarrah K, Packirisamy V, Al Anazi N, Nayak SB. Morphometric analysis of foramen magnum and occipital condyle using CT images for sex determination in a Saudi Arabian population. Morphologie. 2022;106(355):260-70. doi: 10.1016/j.morpho.2021.07.006.
72. Atreya A, Shrestha R, Bhandari K, Malla SK, Acharya S, Menezes RG. Morphometric analysis of the foramen magnum in sex estimation: An additional 3DCT study from Nepal on a larger sample. Health Sci Rep-US. 2023;6(1):e999. doi: 10.1002/hsr2.999.
73. Soon LP, Yi-Li W, Noor MHM, Ibrahim MA. Osteometry of foramen magnum by using post-mortem computed tomography (pmct) for discriminant analysis of sex and population affinity among Malaysian population. Arab Journal of Forensic Sciences and Forensic Medicine. 2023;5(1):43-57. doi: 10.26735/EPUO6928.
