# Supplementary material for: Sex estimation techniques based on skulls in forensic anthropology: A scoping review
Source: PLoS One. 2024 Dec 9;19(12):e0311762. doi: 10.1371/journal.pone.0311762 (PMC11627412; doi:10.1371/journal.pone.0311762)
Supplement: Attachment — Submitted filename: Comments-PONE-D-24-32582.docx [file pone.0311762.s003.docx]

Thank you for the opportunity to review this interesting article. I have a few questions, which are listed below:

The present study is a very elaborative, systematic review of sex estimation techniques based on skulls in Forensic Anthropology. Now my query is what contribution it has made in the identification process. Please add one paragraph related to it.

Page no. 10, 1st Paragraph, line no. 195: Remove the in from “resulting in in” is written twice.

Page no. 10, Modalities: please specify why CT is the most preferred imaging modality.
